# Degradation of FAK-targeting by proteolytic targeting chimera technology to inhibit the metastasis of hepatocellular carcinoma

**DOI:** 10.32604/or.2024.046231

**Published:** 2024-03-20

**Authors:** XINFENG ZHANG, SHUANG LI, MEIRU SONG, YUE CHEN, LIANGZHENG CHANG, ZHERUI LIU, HONGYUAN DAI, YUTAO WANG, GANGQI YANG, YUN JIANG, YINYING LU

**Affiliations:** 1 The PLA 307 Clinical College of Anhui Medical University, The Fifth Clinical Medical College of Anhui Medical University, Hefei, 230032, China; 2 Liver Tumor Diagnosis and Research Center, 5th Medical Center of the PLA General Hospital, Beijing, 100039, China; 3 Department of Infection Diseases, Guizhou Medical University, Guiyang, 550025, China; 4 302 Clinical Medical School, Peking University, Beijing, China; 5 Cell and Gene Therapy Innovation Center, Beijing Lotuslake Biomedical, Science and Technology Park, Beijing, 102206, China; 6 State Key Laboratory of Chemical Oncogenomics and the Institute of Biopharmaceutical and Health Engineering (iBHE), Shenzhen International Graduate School, Tsinghua University, Shenzhen, 518055, China

**Keywords:** Hepatocellular carcinoma (HCC), Focal adhesion kinase (FAK), Proteolytic targeting chimera technology (PROTAC), Epithelial-mesenchymal transformation (EMT), Metastasis

## Abstract

Liver cancer is a prevalent malignant cancer, ranking third in terms of mortality rate. Metastasis and recurrence primarily contribute to the high mortality rate of liver cancer. Hepatocellular carcinoma (HCC) has low expression of focal adhesion kinase (FAK), which increases the risk of metastasis and recurrence. Nevertheless, the efficacy of FAK phosphorylation inhibitors is currently limited. Thus, investigating the mechanisms by which FAK affects HCC metastasis to develop targeted therapies for FAK may present a novel strategy to inhibit HCC metastasis. This study examined the correlation between FAK expression and the prognosis of HCC. Additionally, we explored the impact of FAK degradation on HCC metastasis through wound healing experiments, transwell invasion experiments, and a xenograft tumor model. The expression of proteins related to epithelial-mesenchymal transition (EMT) was measured to elucidate the underlying mechanisms. The results showed that FAK PROTAC can degrade FAK, inhibit the migration and invasion of HCC cells *in vitro*, and notably decrease the lung metastasis of HCC *in vivo*. Increased expression of E-cadherin and decreased expression of vimentin indicated that EMT was inhibited. Consequently, degradation of FAK through FAK PROTAC effectively suppressed liver cancer metastasis, holding significant clinical implications for treating liver cancer and developing innovative anti-neoplastic drugs.

## Introduction

HCC is one of the most common malignant tumors worldwide, ranking third in terms of mortality [1]. Metastasis and recurrence can primarily lead to the high mortality rate in HCC. Late-stage liver cancer can easily metastasize. Among all extrahepatic metastases, the lung is the most common organ for metastasis (38.4%) [2]. Therefore, the control of metastasis in HCC remains a major challenge. Only further exploration of the molecular mechanisms underlying metastasis in HCC and identification of relevant targets can provide new therapeutic strategies for HCC. FAK is a member of the non-receptor protein tyrosine kinases (PTKs) subfamily 1. FAK contains four functional domains: FERM, catalytic kinase, three proline-rich domains (PRI, PRII, and PRIII), and focal adhesion targeting (FAT) domain [3]. FAK has two distinct functions: a kinase-dependent function and a non-kinase-dependent scaffold function, both of which are critical in cancer development, early embryonic development, and reproduction [4,5]. Alan Serrels et al. found that FAK promotes tumor cell survival and tumor progression in squamous cell carcinoma (SCC) by inhibiting the immune response and inducing Tregs to inhibit cytotoxic CD8+T cells [6]. However, existing clinical studies have shown that small molecule inhibitors of FAK have little effect in the treatment of malignant tumors. One possible reason is that FAK only acts on one protein kinase domain, which increases the chance of drug resistance or incomplete inhibition of FAK [7]. Consequently, it is still necessary to develop more effective drugs targeting FAK.

The protein degradation technique, also known as proteolytic targeting chimera (PROTAC), is a new approach to studying post-translational protein modification [8]. PROTAC is a heterofunctional small molecule with two recognition components, one of which specifically binds to E3 ubiquitin ligase, and the other specifically binds to a target protein via an adhesive. PROTAC drives E3 ubiquitin ligase to bind to the target protein, leading to ubiquitination and subsequent proteasome-mediated degradation of the target protein [4]. Therefore, Gao et al. designed and synthesized FAK PROTAC containing an FAK inhibitor (PF562271) and CRBN E3 ligand. FAK PROTAC, named FC-11, leads to rapid and reversible degradation of FAK at dose concentration 50 nM (DC50) in various cell lines *in vitro*, suggesting that FAK PROTAC can be used as an expanded tool and a potential therapeutic agent to investigate the function of FAK in biological systems [9,10].

This study first investigated the effect of FAK PROTAC on degrading FAK in HCC cells and used FAK PROTAC and phosphorylation inhibitors to measure their effects on cell proliferation and function. Then, we measured their efficacy in a lung metastasis model of HCC. The results showed that compared with the inhibition of FAK kinase, FAK PROTAC directly degraded FAK to effectively inhibit the invasion and metastasis of HCC cells.

## Materials and Methods

### Cell culture

In this study, HepG2, MHCC97-H, Hep3B, and Huh7 cell lines were cultured in DMEM or MEM medium (EallBio, Beijing, China) supplemented with 10% fetal bovine serum (Viva Cell, Shanghai, China), 100 U/mL penicillin and 100 mg/mL streptomycin (Thermo Fisher Scientific, Waltham, MA, USA) in a 37°C humidity incubator with 5% CO_2_. The cell lines preserved in our research group were used in this study. All cell lines were verified by STR profiling. Defactinic, an FAK phosphorylation inhibitor, was purchased from MedChemExpress [11] (MCE, Shanghai, China), and FAK PROTAC (FC-11) was produced by MOE Key Laboratory of Protein Sciences, School of Pharmaceutical Sciences, MOE Key Laboratory of Bioorganic Phosphorus Chemistry & Chemical Biology, Tsinghua University.

### Cell proliferation

Hep3B and Huh7 cells were seeded at a density of 5 × 10^3^ cells/well in a 96-well plate. Cells were cultured in DMEM or MEM medium supplemented with 10% fetal bovine serum, 100 U/mL penicillin, and 100 mg/mL streptomycin and kept in an incubator at 37°C with 5% CO_2_. After cell incubation and full attachment, the serum-free medium was replaced. Add their respective effective concentrations, DMSO (1‰) (EallBio, Beijing, China), Defactinib (1 μM), or FAK PROTAC (FC-11, 100 nM) were added according to the experimental plan. Cell counting kit-8 (CCK-8; Dojindo Laboratories, Japan) solution was added to each well at 12, 24, 36, and 48 h of seeding based on the experimental plan and the manufacturer’s protocol. Then, after incubation for 2 h, absorbance was measured at 450 nm using a microplate reader, and values were normalized to the control condition. Each experiment was independently repeated three times.

### Cell migration assay

Hep3B and Huh7 cells were seeded in 6-well tissue culture plates at a density of 4 × 10^5^ and 2 × 10^5^ cells/well, and cultured for 48 and 24 h, respectively, until reaching confluence. After serum starvation for 12 h, scratch wounds were generated in the center of each well using a 200 µL pipette tip. Cells were washed with 2 mL PBS (Huaxingbio, Beijing, China) to remove any debris and then treated with DMSO (1‰), defactinib (1 μM), or FAK PROTAC (FC-11, 100 nM) in supplemented MEM or DMEM medium. Cell migration was monitored by capturing images at 0, 12, 24, 36, and 48 h with an Olympus microscope under 10× magnification. The scratch wound areas were measured at least 5 times at different locations using ImageJ. The average scratch wound area was calculated for each well. Each experimental condition was independently repeated three times. Data were analyzed using appropriate statistical methods.

### Cell invasion assay

To assess cell invasion, a Boyden chamber (6.5 mm Transwell with 8.0 µm pore size polycarbonate membrane, sterile) was utilized in this study. HEP3B and Huh7 cells were harvested, counted using trypsin, and resuspended in a serum-free medium at a concentration of 200,000 cells. In total, 800 µL of medium containing 20% fetal bovine serum was added to the lower chamber, and 100 µL of resuspended cells were added to the upper chamber. The mixture was incubated at 37°C and 5% CO_2_ for 48 h. After the incubation, the medium in the upper chamber was removed, and cells were fixed with paraformaldehyde and stained with 1% crystal violet (Huaxingbio, Beijing, China). Images of invaded cells were captured using a Nikon inverted microscope at 20× magnification. Three independent experiments were performed for each condition.

### Immunohistochemistry

Immunohistochemistry was performed to examine the expression of FAK, phospho-FAK Tyr397, E-cadherin, and vimentin in liver tissue sections. The following antibodies were used: rabbit monoclonal E-cadherin (Cell Signaling Technology, Danvers, MA, USA, 3195S, 1:200), and rabbit monoclonal vimentin (Abcam, Cambridge, MA, USA, ab92547, 1:200). After dehydration and rehydration of liver tissue sections, antigen retrieval was performed by heating the sections in a microwave for 10 min at 95°C. Endogenous peroxidase activity was blocked by incubating the slides with 3% H_2_O_2_ for 10 min. The samples were incubated with primary antibodies at 4°C overnight. The sections were then incubated with appropriate secondary antibodies (biotinylated goat anti-rabbit IgG, 1:200; anti-goat IgG antibody, 1:200) for 40 min at room temperature, followed by detection with 3,3′-diaminobenzidine as the chromogen. A negative control was included for each antibody by substituting the primary antibody with PBS. Three independent experiments were performed for each assay.

### Western blotting

Western blotting was performed to measure the expression of FAK, phospho-FAK Tyr397, E-cadherin, vimentin, and β-actin in cell lysates. The following antibodies were used: rabbit polyclonal FAK (Invitrogen, Carlsbad, CA, USA, PA5-17591, 1:1000), rabbit monoclonal phospho-FAK Tyr397 (Invitrogen, Carlsbad, CA, USA, 700255, 1:1000), rabbit monoclonal E-cadherin (Cell Signaling Technology, Danvers, MA, USA, 3195S, 1:1000), rabbit monoclonal Vimentin (Abcam, Cambridge, MA, USA, ab92547, 1:1000), and rabbit polyclonal β-actin (Proteintech, Wuhan, Hubei, China, 81115-1-RR, 1:5000).

Cells were lysed in sample buffer supplemented with 100 mM dithiothreitol. Cell lysates were heated at 95°C for 5 min, and then centrifuged at 12,000 RPM for 10 min to remove cell debris. The samples were separated by SDS-PAGE (Huaxingbio, Beijing, China) and transferred to cellulose nitrate membranes. After blocking with 5% BSA (EallBio, Beijing, China), the membrane was incubated with diluted primary antibodies at 4°C overnight. The membrane was washed three times with TBST for 10 min each time. Then, the samples were incubated with HRP-conjugated secondary antibody (Solarbio, Beijing, China) at room temperature for 1 h. ECL hypersensitivity (NCM Biotech, Suzhou, China) was used, and signal visualization was performed using the chemidocxrs imager (Bio-Rad, Hercules, CA, USA, #170-8265). Β-actin was used as the loading control. Three independent experiments were performed for each condition.

### Animal experiments

Male BALB/c-nu/nu nude mice (6 weeks old) purchased from Spife (Beijing, China) Biotechnology Co., Ltd. All experimental procedures strictly complied with the Institutional Animal Care and Use Committee (IACUC) guidelines and were approved by the IACUC of Chinese PLA General Hospital (IACUC-approved protocols #2023-0002). All mice were housed in standard conditions with 12-h light/dark cycles and had free access to food and water. Approximately 3 × 10^6^ Huh7 cells were injected intravenously into each mouse. Male nude mice injected with tumor cells were randomly divided into 3 groups (n = 5 in each group): (1) oral carrier twice a day and intraperitoneal injection of PBS (control); (2) Defactinib 25 mg/kg orally, twice daily; (3) FC-11 20 mg/kg, intraperitoneally injected twice a day. After 6 weeks, all surviving mice were euthanized by cervical dislocation under deep anesthesia, and their livers and lungs were dissected and fixed with buffered neutral formalin. Standard histological examination was performed by H&E staining.

### Database query

To investigate FAK gene changes and expression in HCC, we conducted a database search using TCGA (https://portal.gdc.cancer.gov), GEPIA (http://gepia.cancer-pku.cn/index.html), and HCCDB (http://lifeome.net/database/hccdb/search.html). We downloaded and sorted RNAseq data of the STAR process from the TCGA-LIHC project. Data and clinical information were extracted in TPM format. The expression levels were visualized using box plots and analyzed using Cox regression. Prognostic data were obtained from Liu et al. [12], an integrated TCGA pan-cancer clinical database providing high-quality survival outcome analytics. In the GEPIA database, we analyzed gene copy numbers for patients with HCC and compared them with other cancer types in the database. In the HCCDB database, we selected HCC patients from three cohorts: liver cancer, cirrhosis, and normal liver, and visualized expression using a scatter plot.

### Statistical analysis

We used ImageJ to analyze all the results of the migration assay, invasion test, Western blotting, and immunohistochemistry. We performed statistical analyses using one-way analysis of variance (one-way ANOVA), Student’s *t*-test, and two-way analysis of variance (two-way ANOVA). The results are expressed as mean ± standard deviation. A *p-*value of less than 0.05 was considered statistically significant. GraphPad Prism 9 (GraphPad Software, Inc.) was used for statistical analysis and drawing graphs.

## Results

### FAK is highly expressed in liver cancer, and its expression is positively associated with poor prognosis

**Figure 1:**
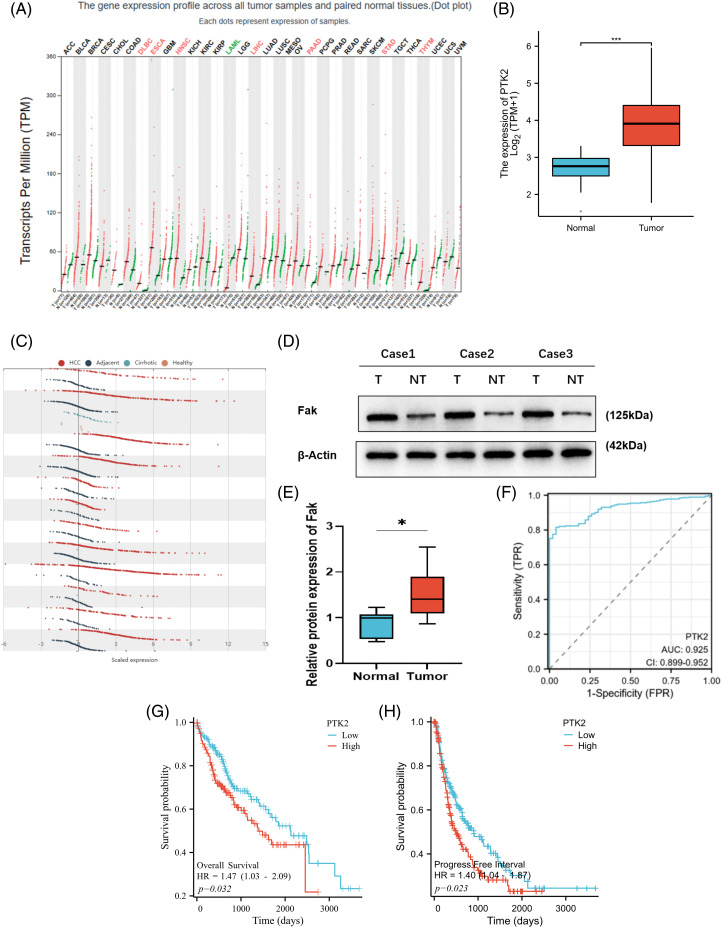
PTK2 is highly expressed in HCC, and its expression is associated with the poor survival of HCC patients. Copy number of PTK2 was significantly increased in HCC compared with other cancer types in the GEPIA of the Pan-cancer genome atlas database. (A) PTK2 copy number was shown in the TCGA database. FAK expression was significantly higher in HCC than in non-cancer tissues. (B) Analysis of HCC, paracancer tissue, and liver cirrhosis samples in the HCCDB showed that FAK was significantly upregulated in HCC compared with liver cirrhosis or paracancer tissue samples. (C) In human HCC and paracancer tissue samples, FAK was significantly higher in tumor tissue than in adjacent normal tissue (*p* < 0.05). (D, E) According to the ROC curve of FAK prediction analysis. (F) The AUC of prediction performance was 0.925, and the 95% confidence interval was (0.899, 0.952). Survival analysis of HCC patients by Kaplan-Meier: In 373 patients with HCC, high FAK expression was negatively correlated with poor overall survival (OS) and progression-free survival (PFS). (G, H) *p < 0.05; **p < 0.01; ***p < 0.001.

Gene profile analysis of the Pan-Cancer Genome Atlas dataset from GEPIA revealed that FAK (PTK2) expression was significantly increased in HCC compared with 31 other cancer types (Fig. 1A). We confirmed these findings by analyzing RNAseq data from the TCGA database, which showed significantly higher FAK copy numbers in HCC compared with non-cancer tissues (Fig. 1B). Further analysis of HCC, paracancer, and liver cirrhosis samples from the HCCDB showed significant amplification or upregulation of FAK in HCC relative to liver cirrhosis or paracancer tissue samples (Fig. 1C). We also observed significantly higher FAK expression in HCC tumor tissue compared with adjacent normal tissue samples (Figs. 1D and 1E). ROC curve analysis showed that FAK is an ideal indicator for HCC, with an AUC value of 0.925% and 95% confidence interval of (0.899, 0.952) (Fig. 1F). Finally, using the TCGA database, we found a negative correlation between high FAK expression and poor overall survival (OS) and progression-free survival (PFS) in 373 patients with HCC (Figs. 1G and 1H).

### FAK PROTAC can effectively degrade FAK

**Figure 2:**
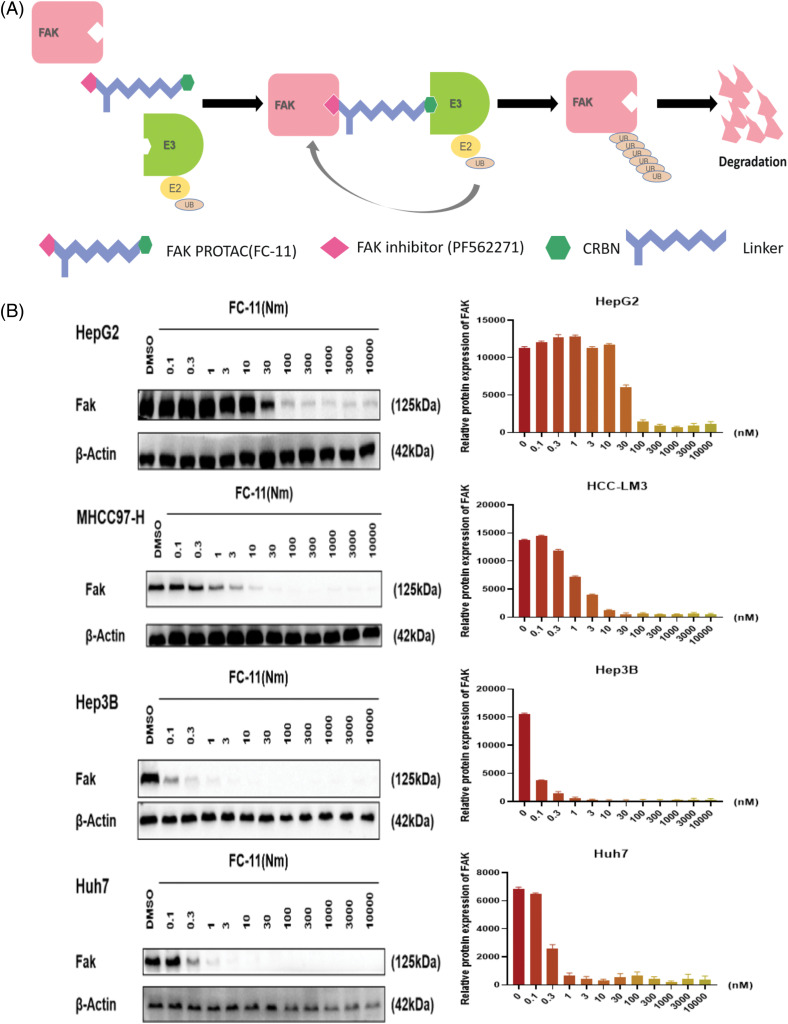
FAK PROTAC effectively degrades FAK. FAK PROTAC binds to FAK kinase inhibitor PF562271 and ubiquitin ligase E3. The mechanism by which FAK PROTAC works is shown in Fig. 2A. (A) Changes of FAK protein in Hep3B, Huh7, MHCC97-H, and HepG2 cells at different doses for 8 h. FAK was completely degraded by FC-11 at a dose of 100 nM. Hep3B and Huh7 were the most sensitive cell lines, and the effective dose was 10 nM (B).

FAK PROTAC (FC-11) was synthesized based on the FAK kinase inhibitor PF562271 (Fig. 2A). Analyzing the degradation efficacy of FC-11 in several HCC cell lines showed significantly reduced FAK levels at a dose of 100 nM, with Hep3B and Huh7 showing the most sensitivity. The effective dose was 10 nM (Fig. 2B). These results indicate that FAK PROTAC can completely degrade FAK, which can be used to investigate the effect of FAK on HCC.

### In vitro, FAK PROTAC more effectively inhibited the migration and invasion of HCC cells than the phosphorylation inhibitor defactinib

**Figure 3:**
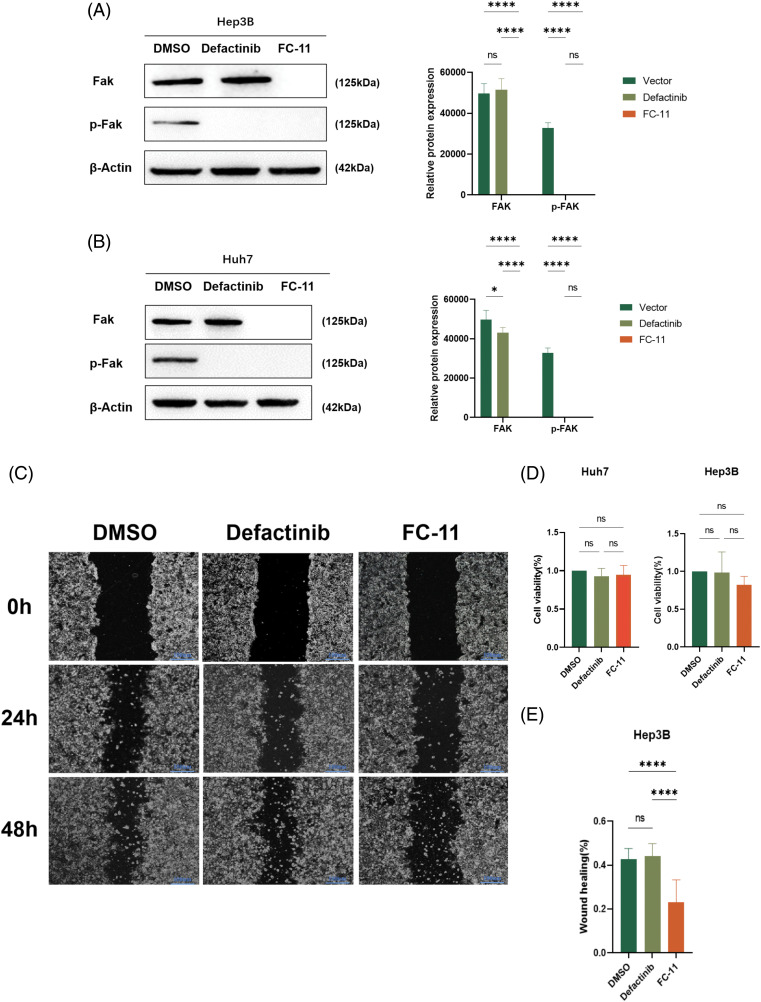

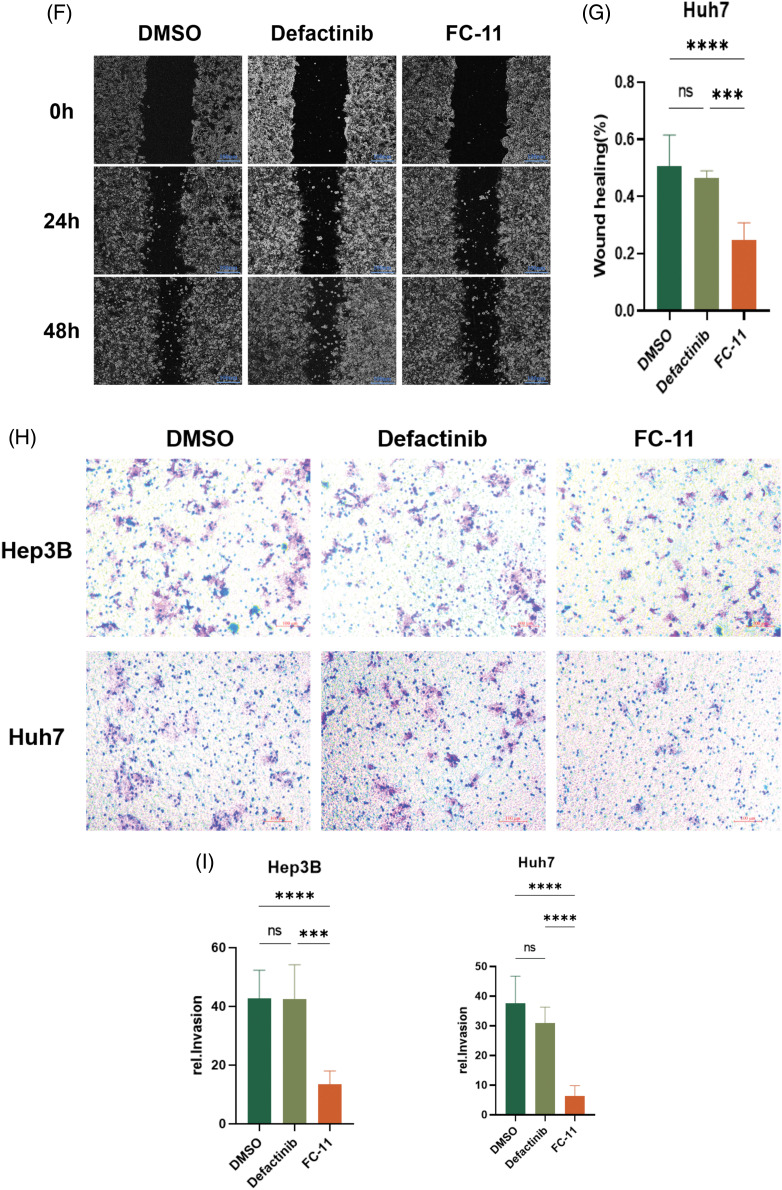
FAK PROTAC is more effective in inhibiting liver cancer cell migration and invasion. Western blotting of FAK and pFAK in Hep3B and Huh7 cells treated with 1 μM defactinib and 100 nM FAK PROTAC (FC-11) for 8 h. (A, B) Cell proliferation was detected by CCK8 assay. Hep3B and Huh7 cells were treated with 1 μM defactinib and 100 nM FAK PROTAC (FC-11) for 48 h. (C) Absorbance and cell viability were measured at 450 nm, and none of them significantly inhibited cell proliferation. Hep3B and Huh7 cells were migrated using wound healing tests. After treatment with 1 μM defactinib and 100 nM FAK PROTAC (FC-11) for 48 h, migration was measure by wound healing experiment at 48 and 0 h. (D–G) The invasion of Hep3B and Huh7 cells was detected. After 48 h of treatmen with 1 μM defactinib and 100 nM FAK PROTAC (FC-11), matrigel-coated transwell plates were used and compared with carrier-treated cells. (H, I) *p < 0.05; ***p < 0.01; ***p < 0.001.

We compared the efficacy of FC-11 and the FAK kinase inhibitor defactinib and found that defactinib inhibited FAK phosphorylation, but it did not reduce the total amount of FAK protein [13]. In contrast, FC-11 not only inhibited the activity of FAK but also completely degraded FAK protein (Figs. 3A and 3B). Additionally, we compared the functional differences between FAK PROTAC and defactinib. Our findings indicated that neither defactinib nor FAK PROTAC (FC-11) significantly inhibited cell proliferation (Fig. 3C). However, wound healing experiments revealed that FC-11 more effectively inhibited cell migration of both cell lines compared with defactinib (Figs. 3D–3G). Similarly, cell invasion experiments demonstrated that FC-11 more effectively inhibited cell migration and invasion of both cell lines compared with defactinib (Figs. 3H and 3I).

### FAK PROTAC inhibits HCC metastasis in vivo

**Figure 4:**
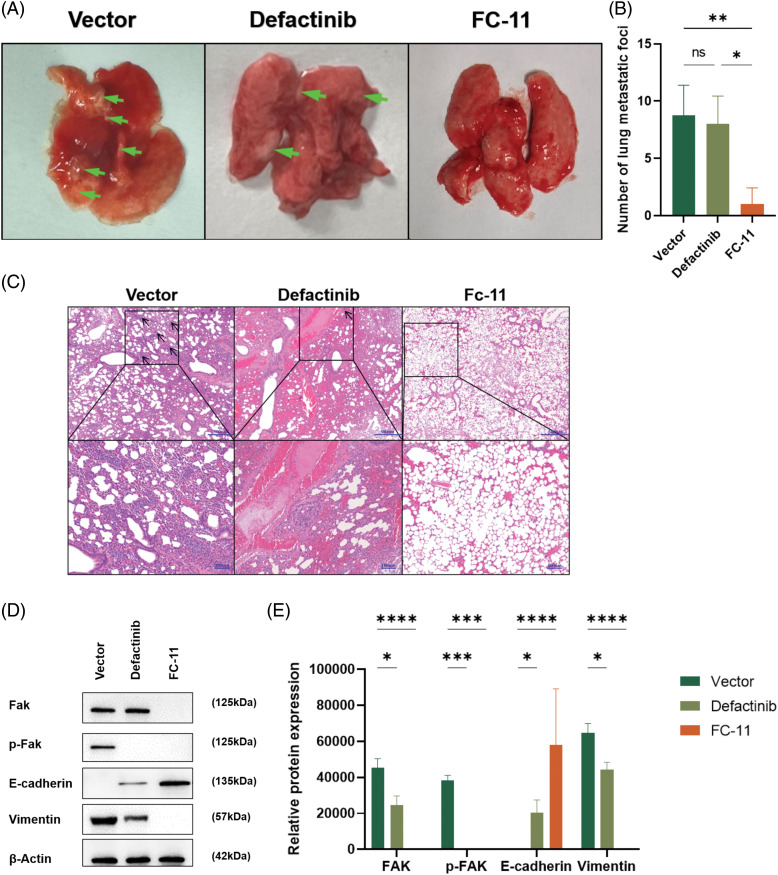
FAK PROTAC inhibits HCC metastasis *in vivo*. Mice were treated with normal saline and intraperitoneal injection, defactinib with 25 mg/kg qd, and FAK PROTAC (FC-11) with 20 mg/kg bid for one week (n = 5 in each group). (A) Representative image of lung metastasis after caudal vein injection in a mouse model. The arrows indicate metastatic nodules. (B) Lung metastasis was detected by H&E staining 6 weeks after intravenous injection of Huh7 cells into nude mice. The number of lung metastases was counted microscopically. (C) Western blotting was used to detect the degradation effect of FAK *in vivo*. We found that FAK was not degraded in the lung metastatic tumor tissues of mice treated with the carrier and defactinib, while it was completely degraded in the tumor tissues of mice treated with FC-11. pFAK was completely inhibited in defactinib and FC-11 groups but not in vector-treated mice. The expression of E-cadherin increased in the FC-11 group, but there was no statistical difference between the other two groups. Vimentin expression was decreased in both defactinib and FC-11 groups, and FC-11 inhibited vimentin expression more than defactinib. (D, E) *p < 0.05; **p < 0.01; ***p < 0.001; ****p < 0.0001.

We found that FAK PROTAC (FC-11) was more effective than defactinib in inhibiting cell migration and invasion *in vitro*. To measure the efficacy of FC-11 *in vivo*, we applied the carrier agent, defactinib, and FC-11 in a lung metastasis model of HCC. The lung metastases of HCC were measured in the animal model after death, and the number of metastases in the FC-11-treated group was less than that in the carrier and defactinib groups (Fig. 4A). After paraffin embedding and H&E staining of the lung tissue, representative images of lung metastatic nodules were obtained under the microscope, and the number of lung metastases was counted. Lung metastasis was significantly inhibited in the FC-11-treated group (Figs. 4B and 4C).

To determine the effect of FAK *in vivo*, we measured FAK content in the above tissues and found that consistent with the results of *in vitro* experiments, FAK did not degrade in tumor tissues of mice in the carrier group and defactinib group. FAK phosphorylation was inhibited in the defactinib and FC-11 groups. FAK was degraded in the FC-11 group (Figs. 4D and 4E).

### FAK PROTAC inhibits EMT of HCC cells by degrading FAK

*In vitro* and *in vivo* experiments have shown that FAK PROTAC affects the invasion and migration of tumor cells by degrading FAK. We investigated whether FAK degradation inhibits HCC metastasis by affecting the EMT of HCC. Previously, we showed that only the phosphorylation of FAK protein, but not its degradation, is inhibited in defactinib-treated lung tissues. In contrast, FC-11 degraded FAK protein compared with the carrier (Fig. 4E). Then, we measured the expression of E-cadherin and vimentin in each group. There was no significant difference in the expression of E-cadherin compared with the carrier-treated group and defactinib-treated group. The expression of E-cadherin was significantly increased, and the expression of vimentin was significantly decreased. Therefore, defactinib and FC-11 both inhibited the phosphorylation of FAK, but only FC-11 degrades FAK and inhibits all functions of FAK, including kinase-dependent pathway and non-kinase-dependent scaffold, upregulates the expression of EMT-related gene E-cadherin, and decreases the expression of vimentin. Therefore, FC-11 can inhibit the EMT and metastasis of HCC cells. In contrast, defactinib only inhibited the activity of FAK and decreased pFAK level, but did not affect the expression of E-cadherin and vimentin in HCC cells, nor could it prevent EMT of HCC cells by inhibiting the phosphorylation of FAK (Figs. 4D and 4E).

### FAK promotes EMT in HCC

**Figure 5:**
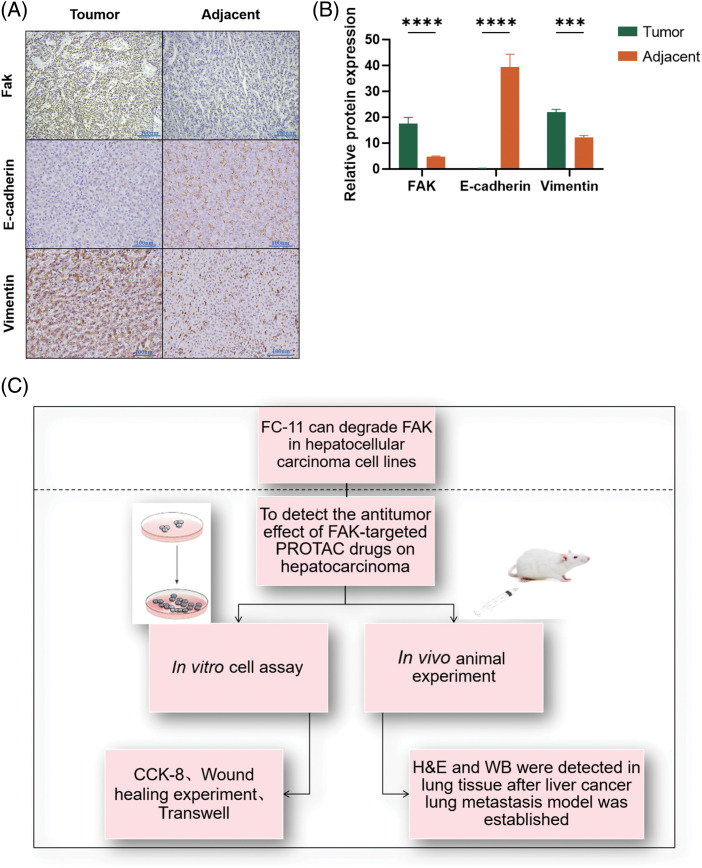
FAK promotes the EMT of HCC. E-cadherin expression was lower, and vimentin expression was higher in liver cancer tissues with high FAK expression compared with paracancer tissues with low FAK expression (*p < 0.05; **p < 0.01; ***p < 0.001; ****p < 0.0001) (A) Action diagram. FAK phosphorylation inhibitors can inhibit FAK phosphorylation. FAK PROTAC inhibited the kinase-dependent and non-kinase-dependent functions of FAK by degrading FAK. (B) Experimental flow chart (C).

To evaluate the role of FAK on EMT, we performed immunohistochemistry and found that the expression of the epithelial marker E-cadherin was significantly lower in cancer tissues with high FAK expression than in adjacent paracancer tissues with low FAK expression (*p* < 0.0001). In contrast, the expression of the mesenchymal marker vimentin was significantly higher in cancer tissues with high FAK expression than in adjacent paracancer tissues with low FAK expression (*p* < 0.001) (Figs. 5A and 5B). These results indicate that FAK promotes EMT in HCC.

Finally, we hypothesized that the role of FAK in promoting EMT may be related to its non-kinase-dependent scaffold function. By comparing the degradation of FAK with FAK PROTAC and phosphorylation inhibitors, we found that FAK PROTAC effectively degraded FAK in HCC cells (*p* < 0.0001), while phosphorylation inhibitors did not significantly affect the expression of FAK or EMT markers. These results suggest that non-kinase-dependent scaffold function of FAK may play a crucial role in promoting EMT in HCC.

## Discussion

We found that FAK PROTAC (FC-11) more effectively inhibited HCC cell migration and invasion than defactinib *in vitro* ( Fig. 5C). *In vivo* experiments using a lung metastasis model of HCC demonstrated that FC-11 inhibited HCC metastasis more effectively than defactinib. FC-11 degraded FAK and inhibited both FAK kinase-dependent and non-kinase-dependent scaffold action. It affected the expression of E-cadherin and vimentin and prevented HCC metastasis by inhibiting EMT.

Previous studies have shown that FAK promotes ovarian cancer proliferation, invasion, and metastasis by interacting with apoptotic peptidase-activating factor 1 (ASAP1) [14]. FAK has multiple phosphorylation sites, most of which are specifically inhibited by ATP competitive antagonists [15]. Clinical trials of GSK2256098, a FAK phosphorylation inhibitor, did not achieve the expected efficacy in treating advanced liver cancer, and its safety also raised some concerns [16]. Therefore, more studies are needed to determine the role of FAK phosphorylation inhibitors in the treatment of HCC. Combination therapy has shown promising results in most cases [17]. FAK deletion inhibited liver cancer metastasis by reducing the expression and activity of matrix metalloproteinase 2 (MMP-2) and matrix metalloproteinase 9 (MMP-9) [18]. FAK is closely related to angiogenesis, and its knockout is embryonically fatal, which greatly hinders further research [19].

With the development of pharmaceutical chemistry, various types of inhibitors, such as PROTACs, have been developed. By degrading FAK, FAK PROTAC inhibits both its kinase and non-kinase functions, providing a tool to explore the non-kinase-dependent role of FAK scaffolds [1]. Unlike previous DNA-based or RNA-based protein knockdown techniques, PROTAC can directly degrade the target protein, making it ideal for functional studies of embryonically fatal proteins in adult organisms. In addition, the strategy allows for precise timing of target protein degradation and reversible recovery of the target protein at a specific time point during the development [20].

FAK is highly expressed in HCC [21], and this study contributes to our understanding of how FAK PROTAC prevents HCC metastasis by ubiquitinating and degrading FAK. FAK degradation affects the expression of E-cadherin and vimentin. E-cadherin and vimentin are key molecules in the invasion of several tumors, such as HCC and colorectal cancer (CRC), playing important roles in tumor cell invasion [22]. High levels of E-cadherin and low levels of vimentin promote HCC invasion. These molecules have become a research hotspot in the diagnosis and treatment of HCC [23,24].

Targeted degradation of FAK by PROTAC not only blocks the kinase activity but also inhibits its non-kinase function as a scaffold protein, showing promising inhibitory effects on tumor cell metastasis and promoting liver cancer cell differentiation and apoptosis.

Consistent with our results, FAK PROTAC (PROTAC-3) developed by Cromm et al. was more effective than defactinib in inhibiting HCC cell migration and invasion [25]. FAK PRTOAC blocks FAK kinase activity and scaffold function by degrading total FAK protein. In terms of mechanism, FAK PROTAC (FC-11) applied in this study was a bifunctional molecule linked by FAK kinase inhibitor PF562271 and E3 ubiquitin ligase (Fig. 2A). It not only inhibited FAK kinase activity by reducing pFAK but also accelerated the ubiquitination and degradation of FAK protease. FAK PROTAC can degrade FAK and inhibit its phosphorylation. Defactinib only blocks FAK kinase activity without damaging FAK protein molecules. FAK PROTAC is more effective than defactinib because it not only disrupts FAK kinase activity by degrading FAK but also disrupts kinase-independent scaffold activity. Therefore, it has been used by researchers as a tool to study the role of FAK scaffolds [25]. In this study, FAK degradation more effectively inhibited EMT than inhibition of phosphorylation. Therefore, it was speculated that its scaffold effect promoted the EMT of tumor cells. The specific FAK inhibitor, PGN-118647, was used to break down FAK at tyrosine 397, resulting in G0/G1 pause, apoptosis, downregulation of enhancer of Zeste homolog 2 (EZH2), and upregulation of Notch homolog 2 (NOTCH2) to inhibit HCC cell growth [26]. However, the inhibition effect was not as effective as that of FAK knockout. Thus, G2/M blockade and subsequent apoptosis caused by FAK knockout may be mainly related to the function of FAK scaffold, necessitating more study on the function of FAK non-kinase-dependent scaffold [6]. These results suggest that the non-classical and classical functions of FAK targeting strategies may interrupt HCC progression. Both irregular and regulated effects of FAK targeting strategies disrupt the progression of HCC. We can explore more possibilities for HCC therapy by targeting the enzyme-dependent and non-enzyme-dependent molecular mechanisms of FAK.

In conclusion, FAK degradation significantly inhibited the lung metastasis of liver cancer compared with inhibition of FAK phosphorylation. The expression of E-cadherin was increased, while the expression of vimentin was decreased, indicating that this drug inhibited liver cancer metastasis. The next step is to study the toxicity, stability, and safety of this drug and use different drug combinations, such as drugs that jointly affect tumor cell proliferation, targeted drugs, immune checkpoint inhibitors, etc., as combination therapy can improve treatment efficacy [1,17].

## Data Availability

The data that support the findings of this study are openly available in TCGA (https://portal.gdc.cancer.gov), GEPIA (http://gepia.cancer-pku.cn/index.html), and HCCDB (http://lifeome.net/database/hccdb/search.html).

## References

[1] Burslem, G. M., Crews, C. M. (2020). Proteolysis-targeting chimeras as therapeutics and tools for biological discovery. Cell*,* 181 *(*1*),* 102–114. 10.1016/j.cell.2019.11.031; 31955850 PMC7319047

[2] Chen, J. S., Huang, X. H., Wang, Q., Chen, X. L., Fu, X. H. et al. (2010). FAK is involved in invasion and metastasis of hepatocellular carcinoma. Clinical & Experimental Metastasis*,* 27 *(*2*),* 71–82. 10.1007/s10585-010-9306-3; 20180147

[3] Cromm, P. M., Samarasinghe, K. T. G., Hines, J., Crews, C. M. (2018). Addressing kinase-independent functions of Fak via PROTAC-mediated degradation. Journal of the American Chemical Society*,* 140 *(*49*),* 17019–17026. 10.1021/jacs.8b08008; 30444612

[4] Cui, L., Zhao, Y., Pan, Y., Zheng, X., Shao, D. et al. (2017). Chemotherapy induces ovarian cancer cell repopulation through the caspase 3-mediated arachidonic acid metabolic pathway. OncoTargets and Therapy*,* 10*,* 5817–5826. 10.2147/ott.S150456; 29263678 PMC5726368

[5] Dawson, J. C., Serrels, A., Stupack, D. G., Schlaepfer, D. D., Frame, M. C. (2021). Targeting FAK in anticancer combination therapies. Nature Reviews Cancer*,* 21 *(*5*),* 313–324. 10.1038/s41568-021-00340-6; 33731845 PMC8276817

[6] de Wispelaere, M., Du, G., Donovan, K. A., Zhang, T., Eleuteri, N. A. et al. (2019). Small molecule degraders of the hepatitis C virus protease reduce susceptibility to resistance mutations. Nature Communications*,* 10 *(*1*),* 3468. 10.1038/s41467-019-11429-w; 31371704 PMC6672008

[7] Gao, H., Wu, Y., Sun, Y., Yang, Y., Zhou, G. et al. (2020). Design, synthesis, and evaluation of highly potent FAK-targeting PROTACs. ACS Medicinal Chemistry Letters*,* 11 *(*10*),* 1855–1862. 10.1021/acsmedchemlett.9b00372; 33062164 PMC7549110

[8] Gao, H., Zheng, C., Du, J., Wu, Y., Sun, Y. et al. (2020). FAK-targeting PROTAC as a chemical tool for the investigation of non-enzymatic FAK function in mice. Protein & Cell*,* 11 *(*7*),* 534–539. 10.1007/s13238-020-00732-8; 32451721 PMC7305269

[9] Gnani, D., Romito, I., Artuso, S., Chierici, M., De Stefanis, C. et al. (2017). Focal adhesion kinase depletion reduces human hepatocellular carcinoma growth by repressing enhancer of zeste homolog 2. Cell Death & Differentiation*,* 24 *(*5*),* 889–902. 10.1038/cdd.2017.34; 28338656 PMC5423113

[10] Guo, D., Song, X., Guo, T., Gu, S., Chang, X. et al. (2018). Vimentin acetylation is involved in SIRT5-mediated hepatocellular carcinoma migration. American Journal of Cancer Research*,* 8 *(*12*),* 2453–2466; 30662803 PMC6325486

[11] He, M., Cao, C., Ni, Z., Liu, Y., Song, P. et al. (2022). PROTACs: Great opportunities for academia and industry (an update from 2020 to 2021). Signal Transduction and Targeted Therapy*,* 7 *(*1*),* 181. 10.1038/s41392-022-00999-9; 35680848 PMC9178337

[12] Jeong, K., Kim, J. H., Murphy, J. M., Park, H., Kim, S. J. et al. (2019). Nuclear focal adhesion kinase controls vascular smooth muscle cell proliferation and Neointimal Hyperplasia through GATA4-mediated cyclin D1 transcription. Circulation Research*,* 125 *(*2*),* 152–166. 10.1161/circresaha.118.314344; 31096851 PMC6702425

[13] Jin, H., He, Y., Zhao, P., Hu, Y., Tao, J. et al. (2019). Targeting lipid metabolism to overcome EMT-associated drug resistance via integrin β3/FAK pathway and tumor-associated macrophage repolarization using legumain-activatable delivery. Theranostics*,* 9 *(*1*),* 265–278. 10.7150/thno.27246; 30662566 PMC6332796

[14] Jin, J., Wu, Y., Chen, J., Shen, Y., Zhang, L. et al. (2020). The peptide PROTAC modality: A novel strategy for targeted protein ubiquitination. Theranostics*,* 10 *(*22*),* 10141–10153. 10.7150/thno.46985; 32929339 PMC7481416

[15] Lim, S. T. (2013). Nuclear FAK: A new mode of gene regulation from cellular adhesions. Molecules and Cells*,* 36 *(*1*),* 1–6. 10.1007/s10059-013-0139-1; 23686429 PMC3887928

[16] Liu, J., Lichtenberg, T., Hoadley, K. A., Poisson, L. M., Lazar, A. J. et al. (2018). An integrated TCGA pan-cancer clinical data resource to drive high-quality survival outcome analytics. Cell*,* 173 *(*2*),* 400–416. 10.1016/j.cell.2018.02.052; 29625055 PMC6066282

[17] Liu, X., Song, X., Zhang, J., Xu, Z., Che, L. et al. (2018). Focal adhesion kinase activation limits efficacy of Dasatinib in c-Myc driven hepatocellular carcinoma. Cancer Medicine*,* 7 *(*12*),* 6170–6181. 10.1002/cam4.1777; 30370649 PMC6308083

[18] Maximin, S., Ganeshan, D. M., Shanbhogue, A. K., Dighe, M. K., Yeh, M. M. et al. (2014). Current update on combined hepatocellular-cholangiocarcinoma. European Journal of Radiology Open*,* 1*,* 40–48. 10.1016/j.ejro.2014.07.001; 26937426 PMC4750566

[19] Popow, J., Arnhof, H., Bader, G., Berger, H., Ciulli, A. et al. (2019). Highly selective PTK2 proteolysis targeting chimeras to probe focal adhesion kinase scaffolding functions. Journal of Medicinal Chemistry*,* 62 *(*5*),* 2508–2520. 10.1021/acs.jmedchem.8b01826; 30739444

[20] Qiu, X., Qiao, F., Su, X., Zhao, Z., Fan, H. (2010). Epigenetic activation of E-cadherin is a candidate therapeutic target in human hepatocellular carcinoma. Experimental and Therapeutic Medicine*,* 1 *(*3*),* 519–523. 10.3892/etm_00000082; 22993570 PMC3445933

[21] Roy-Luzarraga, M., Hodivala-Dilke, K. (2016). Molecular pathways: Endothelial cell FAK–A target for cancer treatment. Clinical Cancer Research*,* 22 *(*15*),* 3718–3724. 10.1158/1078-0432.Ccr-14-2021; 27262114 PMC5386133

[22] Sabri, A., Rafiq, K., Seqqat, R., Kolpakov, M. A., Dillon, R. et al. (2008). Sympathetic activation causes focal adhesion signaling alteration in early compensated volume overload attributable to isolated mitral regurgitation in the dog. Circulation Research*,* 102 *(*9*),* 1127–1136. 10.1161/circresaha.107.163642; 18356543 PMC3092391

[23] Shi, Y., Ge, C., Fang, D., Wei, W., Li, L. et al. (2022). NCAPG facilitates colorectal cancer cell proliferation, migration, invasion and epithelial-mesenchymal transition by activating the Wnt/β-catenin signaling pathway. Cancer Cell International*,* 22 *(*1*),* 119. 10.1186/s12935-022-02538-6; 35292013 PMC8922890

[24] Soria, J. C., Gan, H. K., Blagden, S. P., Plummer, R., Arkenau, H. T. et al. (2016). A phase I, pharmacokinetic and pharmacodynamic study of GSK2256098, a focal adhesion kinase inhibitor, in patients with advanced solid tumors. Annals of Oncology*,* 27 *(*12*),* 2268–2274. 10.1093/annonc/mdw427; 27733373

[25] Wang, B., Li, H., Zhao, X., Zhang, W., Zhao, G. et al. (2021). A luminacin D analog HL142 inhibits ovarian tumor growth and metastasis by reversing EMT and attenuating the TGFβ and FAK pathways. Journal of Cancer*,* 12 *(*18*),* 5654–5663. 10.7150/jca.61066; 34405025 PMC8364639

[26] Xiong, X., Wang, Y., Liu, C., Lu, Q., Liu, T. et al. (2014). Heat shock protein 90β stabilizes focal adhesion kinase and enhances cell migration and invasion in breast cancer cells. Experimental Cell Research*,* 326 *(*1*),* 78–89. 10.1016/j.yexcr.2014.05.018; 24880126 PMC4120946

